# Inflammatory Bowel Disease as a Risk Factor for Premature Coronary Artery Disease

**DOI:** 10.14740/jocmr2102w

**Published:** 2015-02-09

**Authors:** Phillip Ruisi, John N. Makaryus, Michael Ruisi, Amgad N. Makaryus

**Affiliations:** a Rhode Island Hospital/Brown University School of Medicine, Providence, RI 02903, USA; b North Shore-Long Island Jewish Health System, Manhasset, NY 11030, USA; c Mount Sinai Beth Israel, New York, NY 10003, USA; d North Shore-Long Island Jewish Health System, Department of Cardiology, NuHealth, Nassau University Medical Center, East Meadow, NY 11554, USA

**Keywords:** Inflammatory bowel disease, Coronary artery disease, Crohn’s disease, Ulcerative colitis

## Abstract

**Background:**

Crohn’s disease and ulcerative colitis are both systemic chronic diseases that alter bowel physiology. The central process in inflammatory bowel disease (IBD) and the associated manifestations are the result of B-cell production of IgG autoantibodies directed against self-antigens in various organ systems including coronary endothelium. Previous studies have demonstrated significant micro-vascular endothelial dysfunction in patients with IBD compared to patients not affected by the disease. We sought to analyze the relation, if any, between IBD and the development of premature coronary artery disease (CAD).

**Methods:**

We queried our hospital database to find IBD patients admitted to the hospital from January 1, 2007 to December 31, 2008. Patients with traditional cardiovascular (CV) disease risk factors including hypertension, congestive heart failure (CHF), diabetes, age ≥ 65, hyperlipidemia, family history, end-stage renal disease (ESRD), and greater than five pack-year smoking history were excluded from the study cohort. The charts of the remaining 300 patients with diagnosed IBD were then analyzed for the incidence of CV disease events including acute myocardial infarction (MI), unstable angina, positive stress testing, and any cardiac intervention including coronary angioplasty and/or intracoronary stent implantation.

**Results:**

Of the 300 patients included, only one patient had a CV disease event. This patient had a positive exercise stress thallium test. Otherwise, the remaining 299 patients (99.7%) did not have any reported CV disease events over the 2-year follow-up period.

**Conclusion:**

Most of the clinical sequelae of CV disease events are the result of inflammatory changes at the vascular level. While IBD is associated with a chronic inflammatory state as reflected by high sedimentation rates, C-reactive protein (CRP), homocysteine levels, etc., our data seem to indicate that chronic inflammation in the absence of traditional risk factors is not associated with an increased risk of premature CV disease events. More wide-scale prospective studies should be performed to elucidate the relationship, if any, between chronic inflammation and CV disease risk.

## Introduction

The term “inflammatory bowel disease (IBD)” refers to two primary diagnoses: Crohn’s disease and ulcerative colitis. Both conditions are chronic inflammatory disease states associated with a relapsing and remitting course with respect to disease activity and symptomatology. The hallmark of both is a state of chronic inflammation involving mainly the gastrointestinal system with the potential for diffuse systemic involvement. In North America, incidence rates range from 2.2 to 19.2 cases per 100,000 person-years for ulcerative colitis and 3.1 to 20.2 cases per 100,000 person-years for Crohn’s disease. While the incidence of ulcerative colitis has largely remained the same over the last 100 years, the incidence of Crohn’s disease continues to rise [1, 2].

Given the chronic inflammatory state of both diseases as evidenced on laboratory studies by elevated serum levels of inflammatory markers such as C-reactive protein (CRP) and homocysteine, along with well-known extra-intestinal inflammatory manifestations, one may postulate the association of IBD with coronary inflammation and subsequent plaque rupture and/or erosion leading to acute coronary syndromes (ACS). Some of the extra-intestinal inflammatory conditions such as migratory arthritis, anterior uveitis, erythema nodosum, pyoderma gangrenosum, and others have a clear documented inflammatory infiltrative process on pathology. In addition, Crohn’s disease is associated with the development of calcium oxalate stones due to a lack of free calcium (as a result of fat saponification) to appropriately bind the oxalate within the bowel lumen. This leads to excessive free oxalate reabsorption from the bowel, which then crystallizes, with serum and urine calcium leading to renal calculi and an increased overall calcium oxalate production. This pathway, in addition to a high inflammatory state, may as well provide the milieu for coronary artery disease (CAD)/ACS [3, 4].

In this study of 300 IBD patients we theorized that the chronic high inflammatory states of these patients would provide an accelerated mechanism for premature CAD and/or ACS. We assessed the correlation, if any, between the presence of documented IBD and CAD events [5, 6].

## Methods

We queried our hospital database (North Shore University Hospital) to find IBD patients admitted to the hospital from January 1, 2007 to December 31, 2008. This query was completed using ICD coding to identify patients with a pre-existing diagnosis of IBD and/or IBD diagnosed during that specific admission. Patients with traditional CV disease risk factors including hypertension, congestive heart failure (CHF), diabetes, age ≥ 65, hyperlipidemia, family history (defined by ATP III guidelines), end-stage renal disease (ESRD), and greater than five pack-year smoking history were excluded from the study cohort. The charts of the remaining 300 patients with confirmed diagnosed IBD with no other traditional CV risk factors were then analyzed for the incidence of CV disease events including ACS encompassing STEMI, NSTEMI, and unstable angina, positive stress testing, and any cardiac intervention that included coronary angioplasty and/or intracoronary stent implantation over the 2-year follow-up period.

## Results

Basic demographic data were not obtained for this patient cohort other than that applied in the methods section since selection was performed via ICD coding only. Of the 300 patients included, only one patient had a CV disease event. This patient had a positive exercise stress thallium test. This one patient demonstrated a reversible perfusion defect on myocardial perfusion imaging that was consistent with ischemia. Otherwise, the remaining 299 patients (99.7%) did not have any reported CV disease events over the 2-year follow-up period. Table 1 [5-9] lists previous similar studies that have investigated the correlation between IBD and CAD. These studies demonstrated variable and conflicting results. In essence, the standard mortality ratios have not shown an increase in CV mortality in patients with IBD, but other studies have shown an increase in intimal carotid thickness compared to a cohort of non-IBD patients. Intimal carotid thickness has proved to be a marker and reliable surrogate for the existence of clinically significant CAD.

**Table 1:** Correlation Between IBD and CAD

Study	Patient population	Result
Dagli et al [5]	IBD: 40 Healthy patients: 40	IBD patients had a significant increase in carotid intimal media thickness (cIMT) compared to the control group (P = 0.01)
Dorn et al [6]	CD: 4,532 UC: 9,533	SMR = 0.7 - 1.5 (NS) SMR = 0.5 - 1.1 (NS)
Kristensen et al [7]	CD: 4,732 UC: 13,622	Relative risk of myocardial infarction = 1.49 (CI: 1.16 - 1.93) in patients with an active flare and 2.05 (CI: 1.58 - 2.65) in chronic disease state
Yarur et al [8]	CD: 173 UC: 183	Adjusted hazard ratio for developing CAD in IBD group = 4.08 (95% CI: 2.49 - 6.70)
Papa et al [9]	IBD: 52 Healthy patients: 20	cIMT significantly higher in IBD patients (0.63 ± 0.15 mm) compared with controls (0.53 ± 0.08 mm) (P = 0.008)
Ruisi et al (current study)	IBD: 300	SMR = 0.0 (NS) 99.7% of the study patients did not have any reported CV events over the 2-year follow-up period

## Discussion

The inflammatory state of ACS has been well validated in both animal and human models. In addition, the pathophysiological milieu of atherosclerosis itself is a pro-inflammatory state, with monocyte infiltration being one of the precursors of fatty plaque development and eventual rupture. The presence and oxidation of intimal lipid particles leads to an increase in the expression of intimal adhesion molecules, which then facilitate the attachment of monocytes and other inflammatory cells to the endothelium. In addition there is an increase in the production of selectins that further provide anchoring and support of inflammatory cells to the endothelial integrins. Crohn’s disease and ulcerative colitis are both systemic chronic diseases that create disturbances in bowel physiology. The central process in IBD and the associated manifestations are the result of B-cell production of IgG autoantibodies directed against self-antigens in various organ systems such as the bowel, skin, joints/synovium, eye, and coronary endothelium. The exact etiology of IBD remains unclear. Multiple theories have been postulated that include virus mediated autoimmune response, dysbiosis or an imbalance in the gut microbiome or diet related such as a hypersensitivity to cow’s milk antigens, or even bacterial infection related with some variable evidence linking mycobacterium paratuberculosis and paramyxovirus to IBD [10, 11].

Regardless of the etiology the overall pathophysiological effect of systemic inflammation is paramount to understanding the disease manifestations of IBD. IBD patients traditionally have elevated homocysteine, hs-CRP, and ESR levels. These markers of systemic inflammation have been demonstrated to have an association with CV disease. This level of inflammation correlates with enhanced endothelial adhesion of inflammatory cells in response to oxidation of intimal lipid particles. With this understanding one would then postulate that IBD patients would be at higher risk for premature CAD and CV events. The JUPITER trial demonstrated that relatively healthy patients with LDL levels less than 130 but with elevated hs-CRP levels ≥ 2 mg/L had a significant reduction in major adverse CV disease events including death when treated with rosuvastatin. This landmark trial highlighted the potential contribution of inflammation and the treatment of these processes via the pleotropic capability of statins to reduce major CV events including death.

Review of the association between other chronic inflammatory diseases such as rheumatoid arthritis (RA) and systemic lupus erythematosus (SLE), for example, also reveals a potential association between chronic inflammation and an increased risk of CAD events. In a sub-study analysis of RA patients enrolled in the Nurses’ Health Study, Solomon et al reported an increased incidence of myocardial infarction (MI) but not stroke in patients with RA (adjusted relative risk of MI 2.0 (95% confidence interval (CI): 1.23 - 3.29)) [10]. The increased incidence of CV disease events in RA patients independent of traditional CV disease risk factors has been confirmed by several other smaller studies [11, 12]. Other studies have shown a statistically higher incidence of CV disease events in patients with SLE as well, even after adjustment for traditional CV risk factors [13].

The association between IBD and CV disease, however, has proven more elusive. Despite the now accepted correlation between IBD disease activity and the incidence of thromboembolic disease, there are currently conflicting data as to the precise relationship, if any, between these IBD and CVD [14, 15]. A recently published Danish registry study with approximately 20,000 patients revealed an statistically significant increased risk of CV disease events in patients with active IBD flares defined by prescription of pharmacologic therapy (such as corticosteroids or anti-tumor necrosis factor agents). During such periods, the relative risk of MI was 1.49 (CI: 1.16 - 1.93) in patients with an active flare and 2.05 (CI: 1.58 - 2.65) for persistent IBD activity. The relative risk of stroke and CV death were similarly increased while the rates of such events during remission periods were essentially commensurate with the control group [7]. This retrospective analysis, however, failed to incorporate smoking and obesity as CVD risk factors. Similarly, a population-based cohort study in Taiwan demonstrated an increased risk of ACS events in patients with reported symptoms consistent with IBD. In this 13-year study, patients with IBD had a 1.53 hazard ratio of ACS after adjustment for traditional CV disease risk factors. Furthermore, the study also had two other important conclusions, namely, that the incidence of ACS events correlated with the severity of IBD symptoms and that the risk of ACS events was highest in younger patients as well as women, findings that were demonstrated in a meta-analysis performed in the United States [1]. A retrospective longitudinal cohort study with matched controls by Yarur et al showed that IBD patients tended to have a lower incidence of traditional CV disease risk factors but even after adjustment, the hazard ratio for the development of CAD events in the IBD group was 4.08 (95% CI: 2.49 - 6.70), further implying that IBD is an independent contributor to the development of CAD [8].

While such retrospective/registry analyses imply an association, there is a fair amount of contrarian data as well. A biochemical analysis of patients with diagnosed Crohn’s disease using the presence of arterial stiffness and increased levels of lipoprotein-associated phospholipase A2 (Lp-PLA2) as surrogates for the risk of CVD events failed to show an association between IBD and CVD. In this study, patients with IBD actually had lower levels of Lp-PLA2 and there was no statistically significant difference in arterial stiffness as assessed by pulse wave velocity between IBD patients and controls. Crohn’s patients had higher relative arterial stiffness, but this was likely the result of the higher incidence of smoking in this cohort [16]. This result conflicted with a previously published assessment of 32 IBD patients in which IBD was associated with a higher degree of arterial stiffness, although information regarding history of disease burden and smoking history was not included in the analysis [17]. Such conflicting data make arrival at a definitive conclusion regarding IBD and CVD troublesome, as do the observational studies analyzing this association. The largest international meta-analysis on this topic by Fumery et al included more than 200,000 IBD patients culled from 33 studies. This study demonstrated no statistically significant association between IBD and CV mortality [18].

Other observational studies analyzing CVD surrogates provide even further confounding results. Carotid intimal thickness (CIMT) has proven to be a reliable surrogate for the existence of CAD. Small studies have demonstrated an increase in intimal carotid thickness in IBD patients compared to a cohort of non-IBD patients. In 2005 a small prospective study by Papa et al demonstrated significantly higher values of common carotid artery intimal-media thickness (IMT) in IBD patients compared to a control group. This was suggestive that IBD patients may have an increase risk of early atherosclerosis than healthy controls [9]. In a follow-up study to this, Maharshak et al compared measured IMT values between 61 IBD patients and 61 matched control patients. This study found no significant difference in IMT between the two groups and suggests that IBD is not a risk factor for accelerated atherosclerosis [19]. TNF alpha is an additional surrogate marker relating IBD and early atherosclerosis. TNF alpha is cytokine involved in both systemic inflammations and stimulates the acute phase reaction. It is also well known that TNF alpha is crucially involved in the pathogenesis and progression of atherosclerosis. In the vasculature it has significant effects on endothelial and vascular smooth muscle function. A common pharmacologic target in IBD patients is the inhibition of TNF alpha. However there is a paucity of literature on the effects of TNF alpha inhibitors and CV disease in IBD patients. A recent Danish nationwide cohort study in 2013 looked at IBD patient and the incidence of CV events in relation to IBD flares. This study found that IBD patients were at an increase risk of CV events during periods of active disease. The use of TNF alpha inhibitors was one of the markers used to define periods of active disease/flares [20].

Our study is one of the few to directly look for the association of CV disease events (STEMI, NSTEMI, unstable angina, along with ischemia on stress myocardial perfusion testing, and any cardiac intervention that included coronary angioplasty and/or intracoronary stent implantation) in a cohort of patients without any traditional thought of CV disease risk factors other than IBD.

Our study did not demonstrate an association with IBD and premature CV disease events in a 300 IBD patient cohort. This result adds to the general conglomeration of studies implying that the association between chronic inflammation and CV disease events may not be as simple as has been hypothesized. Simply stated, premature CV disease events were not demonstrated in a cohort of IBD patients without traditional CV disease risk factors. It is likely that the systemic inflammatory state alone is not sufficient to generate CV disease events but rather that the chronic inflammation wrought by IBD plays a role in facilitating an already present underlying process such as the intimal lipid particle oxidation in hyperlipidemic patients which is known to be the necessary catalyst for endothelial adhesion molecule production. This then leads to inflammatory cell attachment to the endothelium and the genesis of CAD [20]. What is clear is that more randomized control trials assessing the interplay between these two processes are necessary, and whether active effective therapy to suppress inflammation in these patients could alter the natural progression of CV disease. Such studies must include rigorous patient stratification based upon disease entity and, in particular, disease burden, degree of inflammation, and the mode of treatment employed.
